# Fitness, risk taking, and spatial behavior covary with boldness in experimental vole populations

**DOI:** 10.1002/ece3.8521

**Published:** 2022-02-09

**Authors:** Jana A. Eccard, Antje Herde, Andrea C. Schuster, Thilo Liesenjohann, Tatjana Knopp, Gerald Heckel, Melanie Dammhahn

**Affiliations:** ^1^ Animal Ecology Institute of Biochemistry and Biology University of Potsdam Potsdam Germany; ^2^ Animal Behaviour Faculty of Biology University of Bielefeld Bielefeld Germany; ^3^ Institute of Ecology and Evolution University of Bern Bern Switzerland; ^4^ BioConsult SH GmbH & Co. KG Husum Germany

**Keywords:** animal personality, automated radio telemetry, behavioral type, fitness, home range, *Microtus arvalis*, parentage, reproductive success

## Abstract

Individuals of a population may vary along a pace‐of‐life syndrome from highly fecund, short‐lived, bold, dispersive “fast” types at one end of the spectrum to less fecund, long‐lived, shy, plastic “slow” types at the other end. Risk‐taking behavior might mediate the underlying life history trade‐off, but empirical evidence supporting this hypothesis is still ambiguous. Using experimentally created populations of common voles (*Microtus arvalis*)—a species with distinct seasonal life history trajectories—we aimed to test whether individual differences in boldness behavior covary with risk taking, space use, and fitness. We quantified risk taking, space use (via automated tracking), survival, and reproductive success (via genetic parentage analysis) in 8 to 14 experimental, mixed‐sex populations of 113 common voles of known boldness type in large grassland enclosures over a significant part of their adult life span and two reproductive events. Populations were assorted to contain extreme boldness types (bold or shy) of both sexes. Bolder individuals took more risks than shyer ones, which did not affect survival. Bolder males but not females produced more offspring than shy conspecifics. Daily home range and core area sizes, based on 95% and 50% Kernel density estimates (20 ± 10 per individual, *n* = 54 individuals), were highly repeatable over time. Individual space use unfolded differently for sex‐boldness type combinations over the course of the experiment. While day ranges decreased for shy females, they increased for bold females and all males. Space use trajectories may, hence, indicate differences in coping styles when confronted with a novel social and physical environment. Thus, interindividual differences in boldness predict risk taking under near‐natural conditions and have consequences for fitness in males, which have a higher reproductive potential than females. Given extreme inter‐ and intra‐annual fluctuations in population density in the study species and its short life span, density‐dependent fluctuating selection operating differently on the sexes might maintain (co)variation in boldness, risk taking, and pace‐of‐life.

## Graphical abstract

Individual animals may vary along a pace‐of‐life syndrome from highly fecund, short‐lived, bold, dispersive “fast” types at one end of the spectrum to less fecund, long‐lived, shy, plastic “slow” types at the other end. The underlying life history trade‐off might be mediated by individual risk taking behavior, which was investigated in this study. With a combination of field and laboratory methods, we show that in a species with distinct seasonal life history trajectories, individual differences in risk‐taking behavior have fitness consequences, which may relate to eco‐evolutionary dynamics of phenotypes and life histories in fluctuating population density.

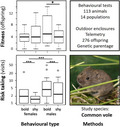

## 1 INTRODUCTION

Risk–reward trade‐offs may favor the coexistence of different behavioral types in populations (Sih & Del Giudice, 2012). Bolder animals may be rewarded for taking higher risks by producing more offspring, and shyer animals may have an increased life span but lower reproductive output per time unit. In species that are highly depredated, however, the fitness gains must clearly outweigh the survival costs of boldness to maintain bold behavior. Alternatively, different behavioral phenotypes can be favored under different environmental conditions, which may lead to similar fitness between behavioral phenotypes and fluctuations of phenotype frequencies in populations (Bergeron et al., 2013; Dingemanse et al., 2004; Nicolaus et al., 2016; Roth et al., 2021).

Similarly, selection related to fluctuations in population density can maintain variation in life history trajectories (Sæther et al., 2016), linking ecological dynamics to evolutionary processes. Life history trajectories may be related to favorable physiological and behavioral phenotypes, forming an extended pace‐of‐life syndrome (POLS, e.g., Careau et al., 2008; Dammhahn et al., 2018; Réale et al., 2010) with fast POL individuals increasing their fitness by higher risk taking, and slow POL individuals by avoiding risks (Wolf et al., 2007a; Wolf et al., 2007b; Wright et al., 2019). Thus, both variation in life histories and the associated among‐individual differences in behavior could potentially be explained through their eco‐evolutionary dynamics with fluctuations in population density (Milles et al., 2022; Wright et al., 2019), group size, or composition of personality in groups (Roth et al., 2019).

Small rodents offer a suitable study system to assess whether and how among‐individual differences translate into variation in risk taking and space use and have consequences for fitness components. Despite extreme predation pressure (Norrdahl & Korpimäki, 1998), consistent individual differences in risk taking have been observed in several small rodent species (Eccard et al., 2020; Herde & Eccard, 2013; Lantová et al., 2011; Mazza et al., 2018). Small mammals in temperate environments follow very distinct life history trajectories within populations, with some individuals—born early or in the middle of the productive season—reproducing immediately and repeatedly in the season of birth and other individuals—born late in the productive season—having to delay maturity, survive the unproductive season and, wait for the next productive season (Eccard & Herde, 2013). These trajectories are flexible and triggered by density‐dependent processes (Prévot‐Julliard et al., 1999), allowing the parallel existence of very different life history trajectories, possibly connected to behavioral differentiation into pace‐of‐life syndromes and maintained by frequency‐dependent selection during density fluctuations (Wright et al., 2019).

Consistent among‐individual differences in behavior may contribute to variation in individual spatiotemporal distribution and might, thus, influence individuals’ interactions with biotic and abiotic components of their environment (Bolnick et al., 2011; Wolf & Weissing, 2010). Whether feedback between space use and individual differences in behavior exists and how this potential feedback drives and/or maintains intraspecific (co)variation in these traits under heterogeneous environmental conditions is matter of current debate (Spiegel et al., 2017). In order to start illuminating these aspects, we need studies quantifying among‐individual differences in behavior and space use independently from each other (e.g., birds; Arvidsson et al., 2017). More ideally, proxies of fitness components, such as reproductive success and survival, would allow assessing the consequences of interindividual differences in behavior. The main aim of this study was to investigate whether between‐individual differences in risk taking and activity behavior that are measured in the laboratory are linked to space use in the field. The second aim was to investigate whether the laboratory measurements can be used to predict survival and reproductive success under field conditions.

We focused on common voles (*Microtus arvalis*), a common microtine rodent, characterized by a high reproductive potential balancing strong predation pressure, a promiscuous mating system (Borkowska & Ratkiewicz, 2010; Fink et al., 2006), larger home ranges of males than females (as other *Microtus* species: Borowski & Owadowska, 2010; Gliwicz, 1997; Solomon & Jacquot, 2011), which are overlapping (Madison, 1980; Spritzer et al., 2006), and male‐biased dispersal (Hahne et al., 2011). Common voles can be concurrently pregnant and lactating and produce litters of 1–8 offspring (median 4–5), depending on mothers’ age (Migula, 1969; Tkadlec & Krejčová, 2001), every 18 days. Parental care is provided by the female alone. As all vole species, common voles are highly depredated by avian and mammalian predators (Halle, 1988; Norrdahl & Korpimäki, 1998; Norrdahl & Korpimӓki, 1995).

To quantify among‐individual variation in two behavioral traits, we conducted two repeated laboratory tests. We measured boldness and activity (Réale et al., 2007), which are highly positively correlated at the phenotypic level in common voles; that is, bolder individuals are more active (Eccard & Herde, 2013; Gracceva et al., 2014; Herde & Eccard, 2013; Lantová et al., 2011). Subsequently, we ecologically validated these personality traits by quantifying space use in a grassland, the natural habitat of common voles, and tested the consequences of among‐individual differences on survival and reproductive success in large outdoor enclosures in experimental populations, which also provided a social environment to the animals. Space use of animals was monitored with automated radio telemetry (ART, e.g., Hoffmann et al., 2018; Kays et al., 2011; Schirmer et al., 2019), and risk taking via radio frequency identification (RFID) systems placed at risky locations. The combination of different methods should allow to complement their respective limitations in temporal or spatial accuracy and detection biases. We tested for relationships between among‐individual differences in boldness on differences in survival probability, risk taking, space use, and reproductive success over 5 weeks under near‐natural conditions. The study period of 5 weeks covers a substantial proportion of an average adult vole's life span of weeks to months (Halle & Stenseth, 2000).

We predicted that individual differences in boldness and activity, quantified in standardized tests in the laboratory, translate into behavioral differences in space use and risk taking under near‐natural conditions. Specifically, we predicted that bold/active individuals occupy larger home ranges and core areas than shy/inactive individuals, as shown for other taxa including birds (Minderman et al., 2010) and small mammals (Boon et al., 2008; Schirmer et al., 2020). Further, we predicted that bold/active individuals—in contrast to shy/inactive individuals—use unsafe open areas at the edge of the suitable habitat patches in large outdoor enclosures (i) with a higher propensity, (ii) a higher frequency, and (iii) longer duration because boldness predicts risk taking (Dammhahn & Almeling, 2012) and dispersal propensity (Cooper et al., 2017) in other small mammals.

We further expected lower survival of bold/active individuals compared to shy/inactive conspecifics because high levels of risk taking and activity may lead to increased predation (meta‐analysis: Smith & Blumstein, 2008; but see Moiron et al., 2020). Elevated exploration and activity might pose a high predation risk, but may result in more encounters with the other sex, or increase attractiveness, and, thus, result in reproductive gains (Ophir et al., 2008; Smith & Blumstein, 2008; Sih et al., 2014; but see Araya‐Ajoy et al., 2016). Contrarily, high activity levels could also be advantageous if they are connected to the speed of exploration, like in eastern chipmunks (*Tamias striatus*) where fast explorers had lower mortality compared to slow explorers, probably because they had increased information about the environment (Bergeron et al., 2013). Our study period included two reproductive cycles of common voles, and we quantified the number of offspring produced during these cycles via genetic parentage analysis. We expected bold/active males to sire more offspring than shy/inactive males. Similarly, we expected bold/active females to have higher reproductive success because they might occupy larger (or better quality) ranges (Schirmer et al., 2019). Moreover, since boldness and exploration correlate in common voles (Herde & Eccard, 2013), bold/active females might provision more food to their offspring than shy/inactive individuals as shown for more explorative blue tit (Mutzel et al., 2013).

## 2 METHODS

### 2.1 Overall study design

Our study had three steps (Table 1): We (a) captured individuals from free‐ranging populations and selected the extreme boldness types to compose experimental populations, (b) released experimental populations of both sexes into near‐natural large outdoor enclosures and recorded space use and risk taking with indirect telemetry methods (ART and RFID) over two reproductive events (5–6 weeks), and (c) recaptured individuals from outdoor enclosures to monitor survival and estimate reproductive success based on parentage analysis. Experimental runs of 14 populations overlapped temporally in part, so that we captured, tested, and monitored space use of animals of different runs in parallel (Table S1).

**TABLE 1:** Schedule of experimental and biological events for common vole subjects in the laboratory and in outdoor grassland enclosures; (a‐c) refer to accompanying sections in the methods part

Experimental time in days	Experimental events	Location
−50	(a) Capture and 3–6 weeks of acclimatization Behavioral testing in cohorts of 24 animals Assembly of experimental populations (b) Application of PITs and radio collars for ART, tissue sampling	Laboratory
−7 to −2		
−1		
−2 to 0	Release of four males each into grassland enclosures Release of four females each into grassland enclosures Exploration phase (Expl) 1^st^ pregnancy of females (Grav1) Parturition of 1^st^ litters, postpartum estrus of females and 2^nd^ mating (Mate) 2^nd^ pregnancy of females while nursing a litter (Grav2) Weaning of first litters (c) Start of removal from enclosures, tissue sampling of first litter (weanlings) for parentage assignment Continuous measurements of movement and risk‐taking behavior	Enclosure
0 to 4		
3 to 15		
16 to 20		
20 to 35		
From 35 on		
−2 to 38		
38 to 40	Parturition of 2^nd^ litters Weaning of 2^nd^ litters, tissue sampling	Laboratory
56		
70	Release to capture locations	

#### 2.1.1 (a) Composing experimental populations

We captured a total of 168 adult common voles between April and August 2010 (*N* = 120) and 2011 (*N* = 48), using baited live traps (Ugglan special No2, Grahnab, Sweden; with shrew exits, Eccard & Klemme, 2013) from meadows around Potsdam, Germany (52°26′21.83″N, 13°00′44.14″O). Animals were housed singly at room temperature 18–23°C and natural seasonal photoperiod in standard rodent cages (Ehret GmbH, Germany, Makrolon Type III: 42 cm × 27 cm × 16 cm). Cages contained pellet food, potatoes, and hay ad libitum, plus wood shavings and paper rolls for shelter. Capture and housing were conducted under permission of the Landesumweltamt Brandenburg (ref. RW‐7.1 24.01.01.10), and experiments were performed under the permission of the Landesamt für Umwelt, Gesundheit, und Verbraucherschutz Brandenburg (LUGV ref. V3‐2347‐44‐2011).

All captured individuals were subjected to a battery of repeated behavioral tests to assess the correlational structure of behavioral variables and temporal consistency of among‐individual differences, that is, animal personality; these results are presented elsewhere (Herde & Eccard, 2013). To compose experimental populations of extreme behavioral types, we used a subset of these individuals and based our selection (see below for more details) on among‐individual differences in activity and boldness quantified in two behavioral tests, the *barrier test* and the *open field test*. All details are in Herde & Eccard, 2013; here, we only briefly describe these test. Both test types were performed in the housing rooms, in small arenas, lasted 5 min each, and were conducted within days (not on the same day). We repeated both test types after two weeks. In the barrier‐test, animals were transferred to one compartment of a two‐compartment plastic box and we measured two behavioral variables: (i) the latency to cross a 2.5 cm barrier into the unknown compartment, and (ii) the frequency of crossing the barrier (expressed as crossings per minute, subtracting the latency to first move). The open field test was performed in a round arena of 1 m diameter, and we measured two behavioral variables: (i) the latency to leave the safe wall zone (defined as a zone of 10 cm width along the wall) and enter the unsafe center zone and (ii) overall activity. In a focal observation of the individual, we recorded via instantaneous sampling and a sampling interval of 10 s whether an individual was active (defined as all types of movement except for cleaning) or inactive, yielding 30 sampling intervals over 5‐min test duration. As detailed in Herde & Eccard, 2013, all behavioral variables were repeatable over time; latencies and activity variables (frequency of crossing and activity in the open field), respectively, were correlated across the two test types; and combined scores for activity and boldness were correlated at the phenotypic level, across all animals tested in the laboratory, male voles were more active than females but sexes did not differ in latencies. The individual time in captivity before the first behavioral test was performed did not explain variation in boldness nor activity (*R* ^2^ < .03, for all four variables).

Behavioral testing started 3–6 weeks after the animals were captured (to ensure that females were not pregnant, and that pregnant females were able to give birth, raise, and wean the litter), and as soon as a cohort of 24 animals had been collected (12 individuals per sex). For logistical reasons, we had to base the selection of individuals for experimental populations on subsequent cohorts of captured individuals. From each test cohort of 24 individuals, we subsampled 16 animals for two experimental populations run in parallel in two enclosures. To select individuals, we ranked same‐sex animals based on the values of behavioral variables obtained in the first test round, that is, according to their latencies (shortest = lowest rank) and activities (most active = lowest rank). From the four behavioral variables, we calculated a mean rank for each individual within its test cohort. The four males and four females with the lowest mean ranks (thereafter called *bold*) and the four males and females with the highest mean ranks (thereafter called *shy*) were used for this experiment and assigned alternatingly by rank to two populations, each consisting of two bold males, two bold females, two shy males, and two shy females. Animals with medium ranks were released at their capture location. Binning into a bold and a shy category was used to be able to compare extreme phenotypes (bold/shy) in replicated populations, reflecting our original hypotheses. Since we had removed intermediate phenotypes from the setup, we refrained from correlating obtained variables to original behavioral values, personality scores, or ranks. This approach potentially limits our ability to detect gradual effects of behavioral phenotypes or specifics of extreme versus intermediate behavioral phenotypes.

Across all test cohorts, absolute values of behavioral variables from individuals classified as *bold* (*n* = 56) differed from those of individuals classified as *shy* (*n* = 56 + 1 one additional shy animal released to the first enclosure by mistake, Student's *t* tests for all behavioral variables 2.4 < *t* < 7.8; all *p* < .02, Tables S1 and S2). Also body weights differed among types, with animals classified as shy being 10% heavier than animals classified as bold (*t* < 2.0, *p* < .049, Table S1).

#### 2.1.2 (b) Experimental populations in large near‐natural grassland enclosures

**FIGURE 1:**
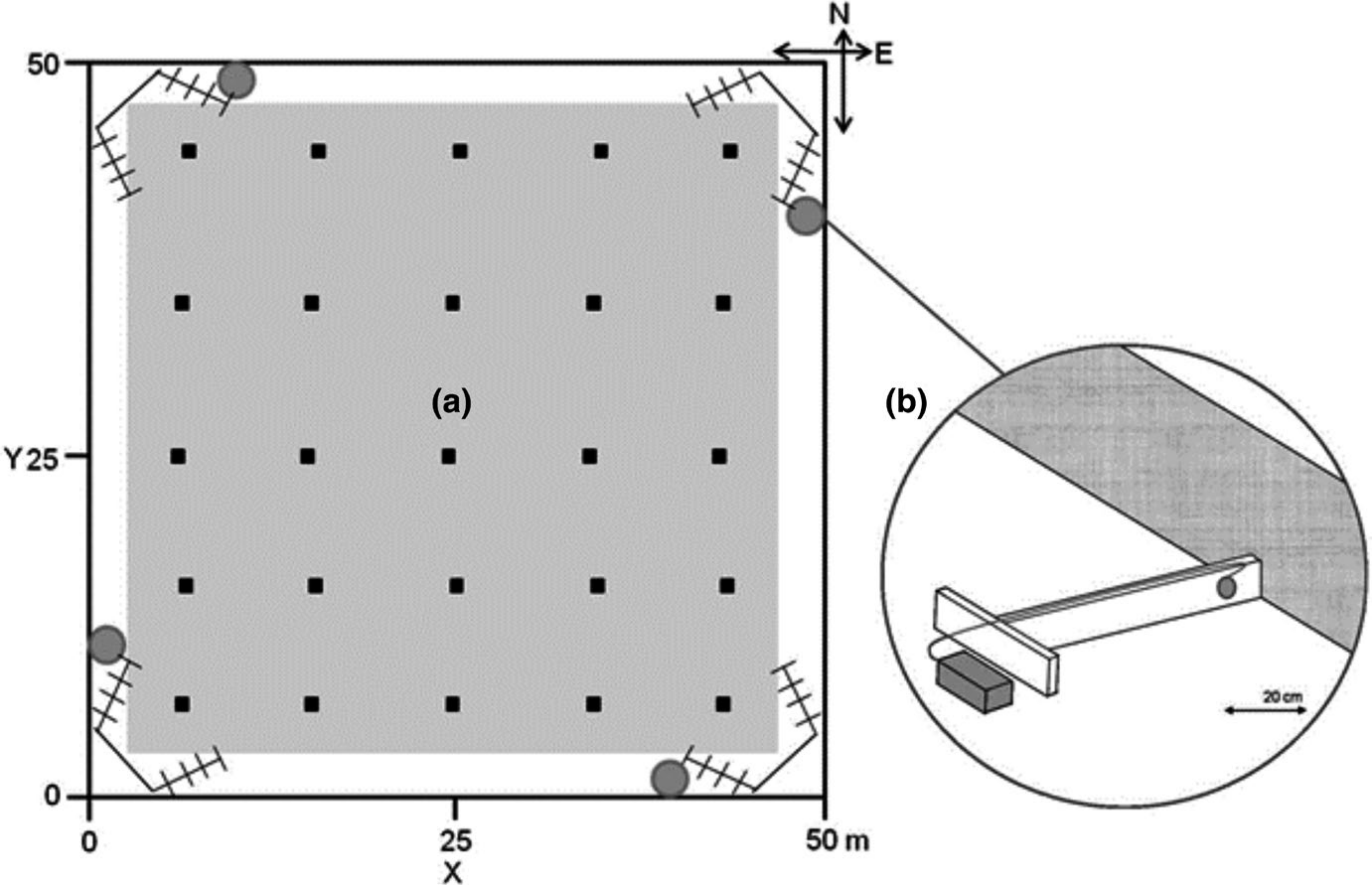
Schematic display of one near‐natural grassland enclosure. (a) Enclosures were equipped with Automated Radio Telemetry (ART) with (1) eight radio telemetry antennae (two in each corner) as part of an automated radio telemetry system (ART), (2) four guided passages with RFID readers (gray circles) in the vegetation free area strip (white area) along the enclosure wall, and (3) 25 live traps (black squares) in vegetation cover (light gray area). (b) Wooden guide passage (white T), wood structure with a hole surrounded by a RFID ring antenna (gray circle) close to the metal wall of the outdoor enclosure (gray surface). Gray box: RFID logger (i.e., storing unit). Note: not drawn true to scale

Experimental populations were kept for five weeks between June and November in one of six large grassland enclosures of 50 m × 50 m each. Each enclosure was fenced with a galvanized metal wall (1 m below and 0.5 m above surface). Enclosures were protected against ground predators by a veterinary fence (2 m height) and an electrical fence, but were open for natural avian predation. Ugglan live traps were set in a regular five by five grid with 10 m distance to recapture individuals at the end of the experiment. Two to four experimental populations were operated in parallel, resulting in 14 population replicates (ten in 2010, four in 2011). Enclosures had a built‐in automated radio telemetry system (ART, Figure 1a), consisting of pairs of four‐element Yagi antennae (Winkler‐Spezialantennen, Germany) mounted on a stand of 3.2 m height in each corner, connected via subterraneous cables to an eight‐channel automated receiving unit for each enclosure (ARU; JDJC Corp., Sparrow systems, Illinois). Vegetation along the inside of the enclosure walls was mowed in a strip of 1.5 m width to prevent animals from climbing (Figure 1a). The strip was kept short by regular hand mowing, and intervals depended on local vegetation height and rain patterns. Mowing did not kill any of the collared voles. In the mowed area, the perceived avian predation risk is high for a ground dwelling mammal (Jacob & Brown, 2000).

#### 2.1.3 (c) Behavior in the field

Before the release into grassland enclosures, each animal was marked with a unique passive integrated transponder (PIT; 0.1g; Trovan ID‐100, Euro ID, Germany), placed subcutaneously in the scapular region, for individual recognition at RFID readers. Animals were fitted with a radio telemetry collar (Biotrack, UK; 1.0 g including cable tie, <5% of mean body mass before release) with an individual radio frequency.

We placed barriers (Figure 1b) perpendicular to the enclosure wall into the vegetation free strip, guiding a passing animal through a passage (diameter 4.5 cm) monitored by an RFID ring antenna (diameter 5.5 cm) with a detection range at ca. 1 cm before and after the ring, and a storing unit (LID 650 Euro ID, Germany). By integrating this system into a drift fence, we assumed to detect individuals that moved in the open areas and along enclosure walls, while short excursions into the open area were not detected. The system recorded each individual RFID code with a time stamp every 0.1 sec. Animals were classified as “visitors” (at least one reading in the edge zone at one of four antennae in 35 days) and “nonvisitors” (no reading). For visitors, we further analyzed *number of visits* (two visits were separated by a minimum of 5 min between two readings) and *mean duration of visits*. The definition of visit bout length was based on a pilot study filming vole behavior at the barriers.

Males were released two days prior to females to display potential differences in exploration behavior and avoid an immediate associate with locations of females (Ims, 1988). Automated Receiving Units (ARU) logged signal strength for each radio tag's frequency on each antenna. In 2010, we logged frequencies in parallel (each frequency once every 2 min) and calculated a location integrating 48 signals across the antenna array over 12 min. After learning from the RFID data in 2010 that individuals could potentially (one animal) cover the length of the enclosure in the duration between two location fixes, we changed the logging rhythm in 2011 to sequential logging; integrating the same number of signals over 1.5 min per animal before switching to the next frequency, while keeping the logging interval of 12‐min constant between years. Within each antennae pair, we converted the distribution of median signal strengths from 12 signals into a bearing. Locations were calculated with triangulation of these bearings (see Hoffmann et al., 2018). We removed data after receiver failures (three populations) or antenna failures (two populations), and during rainy periods within replicates (poor transmission through wet vegetation). Overall, we obtained *N* = 1004 telemetry days with 90–120 locations per day for 58 individuals in nine populations (mean ± SD: 20 ± 10.4 per individual, min‐max range: 3–35 day ranges). We conducted calibration, precision, and maintenance checks on the telemetry grids before, in the middle, and after each replicate, using stationary experimental tags. Calibrations lasted ca. 1 h leaving sufficient time to collect locations for the respective day ranges. We found that the absolute day range sizes varied greatly between populations (ANOVA of 95% Kernels: *F* = 135, *p* < .001, *df* = 8; population means from 348 m² to 1168 m²; data were square‐root‐transformed beforehand), and also the precision of single locations varied between experimental populations (5 to 15 m) due to seasonal changes in vegetation height, wind, or moisture. To account for differences among replicates, we obtained z‐scores within populations, relating the individual day range to the respective population mean (in: percent of population mean). We calculated day ranges (i.e., 95% density Kernels) and core area of day ranges (i.e., 50% density Kernels) as estimates of individual vole movement and mobility (Worton, 1987, 1995). Since all analyses conducted with both kernel sizes yielded very similar statistics (both were based on the same location data set), we present only the statistical results on day ranges here.

We hypothesized that behavior in the field also depends on the social interactions in the experimental population. Particularly, mobility of males may vary with the availability of mating partners, which would be low during synchronized pregnancy phases of females. Further, we hypothesized that boldness types may cope differently with being released to the unknown habitat (Veerbek et al., 1994). Therefore, we included the following experimental phases based on the species’ life history into the analysis of location data (Table 1): (1) Expl: exploration phase (days 0–3), when animals explore the habitat and unknown conspecifics and mating occurs (females were introduced nongravid) resulting in synchronous reproduction cycles; (2) Grav1: the first pregnancy when females were synchronously gravid (days 4–15); (3) Mate: a phase during which females give birth and mate again postpartum (days 15–20) with males presumably increasing mobility to roam between females; and (4) Grav2: a second pregnancy phase where females were supposedly gravid again (days 20–35).

#### 2.1.4 (d) Fitness

To remove adults and their offspring from the enclosures, we set live traps after 35 days (Table 1). It took up to 5 days until animals were removed. Individuals without a mobile radio tracking signal that were not recaptured during removal trapping or did not re‐appear in a later replicate (6 cases) were considered to be dead. To estimate reproductive success, we collected small tissue samples from the ears of adult voles before release to the enclosures, from offspring born in and captured from the enclosures (*N* = 335 juveniles), and from offspring born to females kept in singe cages after the experiment (*N* = 85 juveniles). Laboratory procedures for genotyping followed Braaker and Heckel (2009; Table S3). Microsatellite alleles were determined using GeneMapper^®^ Software, version 3.7 (Applied Biosystems). The number of alleles ranged between two and 32 (mean = 13.9) per microsatellite locus. We used the software *CERVUS* 3.0.3 (Kalinowski et al., 2007; Marshall et al., 1998) and individual parental candidate exclusion for parentage identifications (details in Table S3).

From the analysis, we obtained 421 parental assignments for 276 offspring and identified both parent candidates for 64% of offspring and one parent candidate for an additional 17.4% of offspring. To obtain robust estimates of relative individual reproductive success of a parent within a population, we included replicates with >16 parentages assigned (8 replicates). The excluded replicates had <8 parental assignments, and we excluded assignments to parents outside their original replicates. With these limitations we were able to use data of 346 assignments for 258 offspring to 57 parental candidates. There were six animals among those candidates where no offspring was assigned, but which had been able to potentially sire offspring, as indicated either by their recapture after the experiment (*n* = 3) or the polyphasic activity signature of their radio signals (*n* = 3, see supplemental material for exemplary diagnostic plots).

#### 2.1.5 (e) Statistical analyses

To test whether boldness type explained variation in risk taking, space use, survival, and reproductive success, we used linear or generalized linear mixed effects models (LMM or GLMM) run with the R package “lme4” (Bates et al., 2015). The underlying error distributions were specified as binomial for probabilities (survival, reproduction, visits of risky areas), as Poisson for count variables (number of visits of risky areas, number of offspring) and as Gaussian for continuous variables (home range size, core area size, duration of visits of risky areas). Given a biased distribution as based on visual inspection, we log‐transformed the duration of visits. Models included our predictor boldness type (shy or bold) and sex (male or female) as fixed effects, and their interaction. Further, we included control variables as fixed effects into initial models: the starting month of the replicate (to control for seasonal variation as covariate) as a continuous covariate, and the experimental phase (with four levels, see Table 1 for details) for models on space use only. Experimental phase was specified in interaction with boldness type and sex because we expected space use to vary with the phases of the experiment and in particular with female reproductive activity in our artificially reproductively synchronized populations. As random effect, we included population replicate ID (specified as random intercept) to control for potential variation among replicates such as vegetation height, rain events, or predation pressure. Such external properties could potentially affect the behavior of the entire population. Furthermore, vegetation and weather may affect the quality of our tracking calibration, the recapture success. For exploration of the data, we experimented with different random structures (e.g., including the identity of the enclosure or adding the year as a fixed factor, but population ID as random factor captured this variation). In models of space use and risk taking, we had repeated measurements of individuals and therefore added individual ID as a second random effect to the mixed models.

We assessed model fit visually based on inspections of residual distribution (homogeneity of variances, normal distribution) and calculated conditional and marginal coefficients of determination (*R*²) using the R package MuMIn (Nakagawa & Schielzeth, 2013). Predictors reflecting the experimental setup (boldness type and sex) were always kept in the final model. Based on log‐likelihood ratio tests, control variables, covariates, and interaction of factors were removed if they did not increase the predictive value of the model. For behavioral variables from the field (risk taking and space use), we estimated repeatability over time using the R package rptR (Nakagawa & Schielzeth, 2013, Stoffel et al., 2017), using 1000 simulations to estimate confidence intervals and 1000 permutations to estimate *p*‐values. Analyses were carried out with R, Version 3.0.2 (R Core Team, 2016).

## 3 RESULTS

### 3.1 Behavior in the field

**FIGURE 2:**
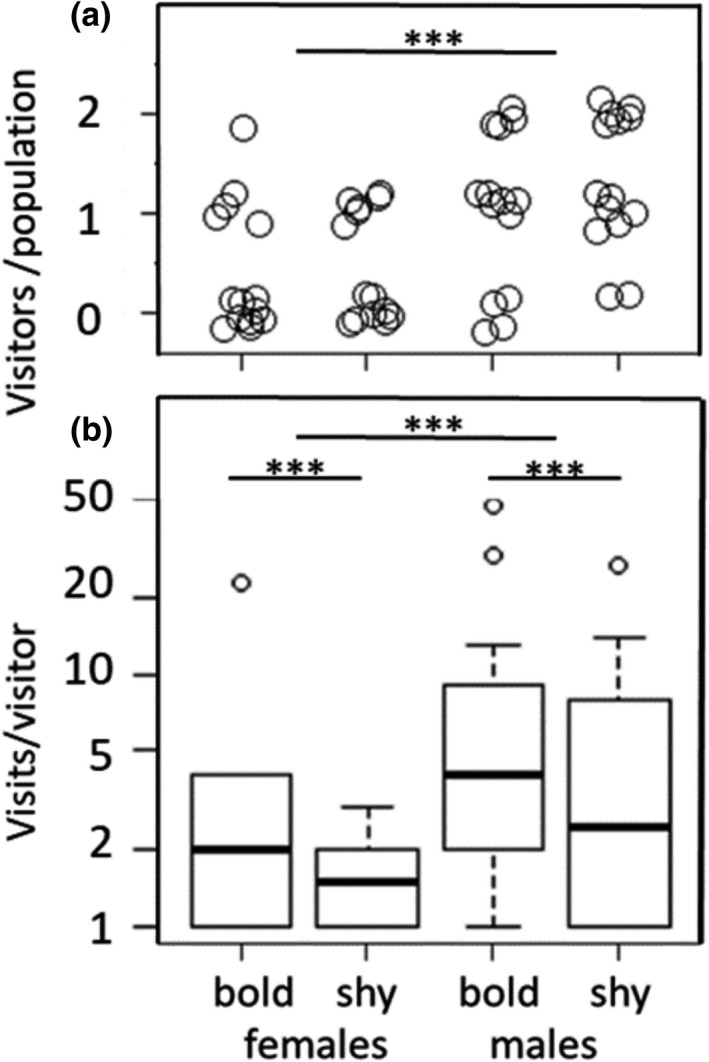
Visits of common voles at the potentially dangerous, vegetation free edge area of the enclosures, shown by sex and boldness type: (a) number of animals visiting per population (*n* = 14 populations, two animals per sex‐boldness type combination per population, jitter plot), (b) number of visits per individual, *n* = 44 visitors, significance levels (***p < .001, *p < .1) Shown are median (line), interquartile range (box), min‐max range (whiskers), and outliers (dots)

**FIGURE 3:**
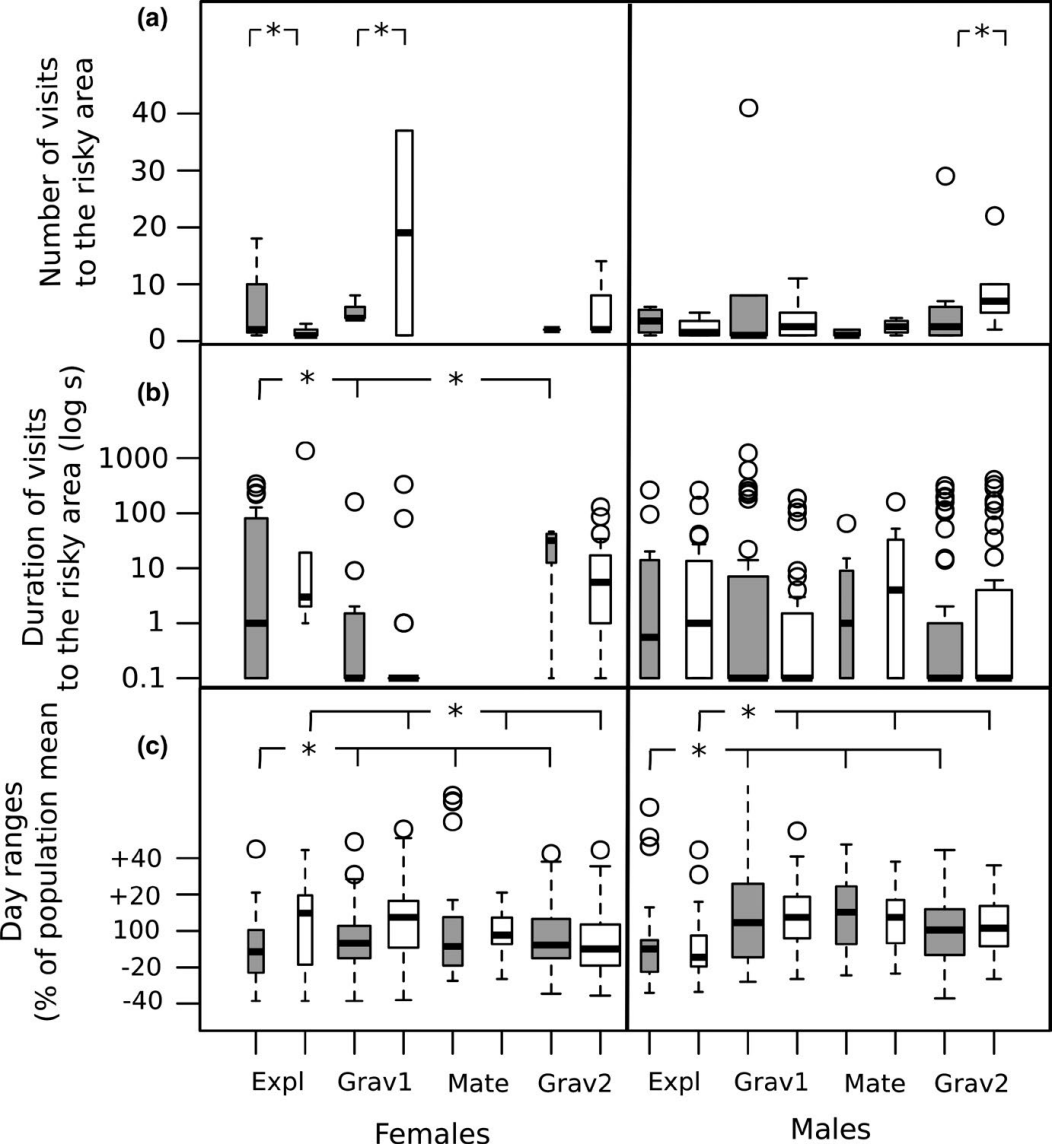
Behavior of common voles in large grassland enclosures over 7 weeks, gray: boldness type “bold,” white: boldness type “shy”. Experiments were divided into four phases based on female reproductive biology: Expl: exploration of novel environment including social environment and mating (3 days); Grav1: first pregnancy (15 days), Mate: parturition of litters and mating after postpartum estrus (5 days), Grav2: second pregnancy (13 days). (a) Number of visits at low vegetation (risky) areas of the enclosures. (b) Duration of visits (*n* = 358 visits by 44 common voles in 14 populations) at low vegetation (risky) areas of the enclosures. Long visits indicate a careful and slow movement at the passage counter, short visits a quick passage. Missing observations: no visits. Width of bar indicates relative sample size. (c) Model predictions for relative size of 1004‐day ranges (95% Kernel estimates) in relation to the respective population mean (= reference line: 100%) for 58 common voles (ID included as random effect) from nine enclosed populations (population included as random effects). Each day range was computed based on 90–120 location fixes per individual over 24 h. Asterisk refers to post‐hoc differences at *p* < .05 (compare Table 3)

Risky areas were visited 367 times by 44 of 113 individuals (39%), which visited at least one guided passage in the vegetation free zones of the enclosures. Females were less likely (yes or no) to visit the risky area (12 of 57 females) than males (32 of 44, χ^2^ = 14.4, *p* < .001, Figure 2a, Table 2) and visitation probability of an animal was independent of its boldness (χ^2^ = 0.4, *p* = .524). Among visiting animals males visited more often (7.4 ± 10 visits) than females (3.5 ± 6.2 visits, χ^2^ = 14.1, *p* < .001, Figure 2b), and bold individuals more often (8.3 ± 11.9) than shy individuals (4.7 ± 6.1 visits, χ^2^ = 22.3, *p* < .001), and the number of visits was higher in replicates later in the season (month: χ^2^ = 4.8, *p* = .029, Table 2). The duration of a visit was highly repeatable within individuals (*R* = .632, CI 0.50–0.73) and was depending on an interaction of experimental phase and sex (χ^2^ = 17.53, *p* < .001, Table 3, Figure 3a). Visited risky areas for shorter periods during the first pregnancy phase (Grav 1) compared with the exploration phase (Expl) and the second pregnancy (Grav 2). In males, we detected no effects of experimental phase or boldness type.

**TABLE 2:** Risk taking (visiting short vegetation areas of enclosures), survival, and reproductive success of common voles in experimental populations

Dependent variable (model, error distribution)		Model estimates					Explained variance	
		Intercept	Boldness type (shy)	Sex (male)	month	Boldness type*Sex	*R* ^2^_marginal_	*R* ^2^_conditional_
Visitation (probability, binomial) *n* = 113, 14 populations	ß ± SE	1.80 ± 0.66	0.26 ± 0.41	**−1.60** ± **0.42**	ns	ns	0.4	0.4
	z	2.7	0.64	**−3.80**				
	*p*	.008	.52	**<.001**				
No. of visits (Poisson) *n* = 44, 14 populations	ß ± SE	1.30 ± 0.30	**−0.60** ± **0.13**	**0.73** ± **0.21**	**0.26** ± **0.11**	ns	0.11	0.4
	z	5	**−4.6**	**3.5**	**2.1**			
	*p*	<.001	**<.001**	**<.001**	**.029**			
Survival (probability, binomial) *n* = 113, 14 populations	β ± SE	4.04 ± 2.18	−0.52 ± 0.44	**−0.74** ± 0.44	**−0.47** ± 0.25	ns	0.25	0.57
	z	1.85	1.17	**1.67**	**1.99**			
	*p*	.064	.224	**.094**	**.06**			
No. of offspring (Poisson) *n* = 57, 8 populations	β ± SE	1.31 ± 0.17	0.22 ± 0.18	**0.37** ± **0.17**	ns	**−0.61** ± **0.25**	0.02	0.12
	z	7.44	1.25	**2.12**		**−2.40**		
	*p*	<.001	.211	**.034**		**.017**		

Note

Shown are model estimates of GLMMs based on different sample sizes of animals (*n*, given for each model) and different numbers of populations, which were included as a random factor. Covariate and interaction were removed if *p* > .1. The reference levels for categorical predictors are bold (for boldness type) and female (for sex). Shown are estimates (β) and their standard errors (SE), z‐values and *p*‐values as well as *R* ^2^_marginal_ as the variance explained by fixed factors, and *R* ^2^_conditonal_ as the variance explained by fixed and random factors. Significant effects are marked with bold font.

**TABLE 3:** Estimates (and their standard error, SE) of effect sizes of sex, boldness type, and experimental phase on behavior of common voles recorded in large grassland enclosures (2500 m^2^) analyzed with linear mixed models. BNT: Boldness type

Variable:	Risk taking			Relative day range size		
Data:	367 visits (log duration (s))			1004 ranges (square root (%))		
Random structure:	44 animals, 14 populations			58 animals, 9 populations		
	Estimate	SE	*t* value	Estimate	SE	*t* value
(Intercept)	2.70	1.34	2.02	22.70	1.72	13.1
Boldness type (shy)	0.07	0.31	0.22	−0.76	1.25	−0.61
Sex (m)	**−1.50**	**0.69**	**−2.18**	**0.05**	**1.38**	**0.03**
PhaseGrav1 (Expl)	**−2.70**	**0.65**	**−4.18**	0.39	0.67	0.58
PhaseMate (Expl)	−0.25	1.45	−0.17	−0.07	0.78	−0.08
PhaseGrav2 (Expl)	−0.08	0.78	−0.10	−1.46	0.69	−2.12
Sex:PhaseGrav1	**2.46**	**0.83**	**2.97**	**2.52**	**0.92**	**2.75**
Sexm:PhaseMate	0.57	1.69	0.34	**2.88**	**1.06**	**2.71**
Sexm:PhaseGrav2	−0.37	0.94	−0.40	**2.53**	**0.93**	**2.71**
BNT:sexm				1.68	1.78	0.94
BNT:phase2grav				1.43	0.94	1.52
BNT:phase3mate				0.24	1.07	0.23
BNT:phase4grav				1.66	0.99	1.68
BNT:sexm:phase2grav				**−2.81**	**1.25**	**−2.25**
BNT:sexm:phase3mate				−1.09	1.45	−0.75
BNT:sexm:phase4grav				**−5.09**	**1.30**	**−3.91**

Note

Risk taking was recorded with RFID readers in risky, short grass areas of enclosures. Range sizes were recorded with automated radio tracking. Random structure corrects for repeats within populations of up to 8 animals, and repeated measures within individuals. Both marginal and conditional *R* ^2^ for risk taking *R* ^2^ = .07, for day ranges marginal *R* ^2^ = .08, conditional *R* ^2^ = .49. Significant effects are marked with bold font.

**FIGURE 4:**
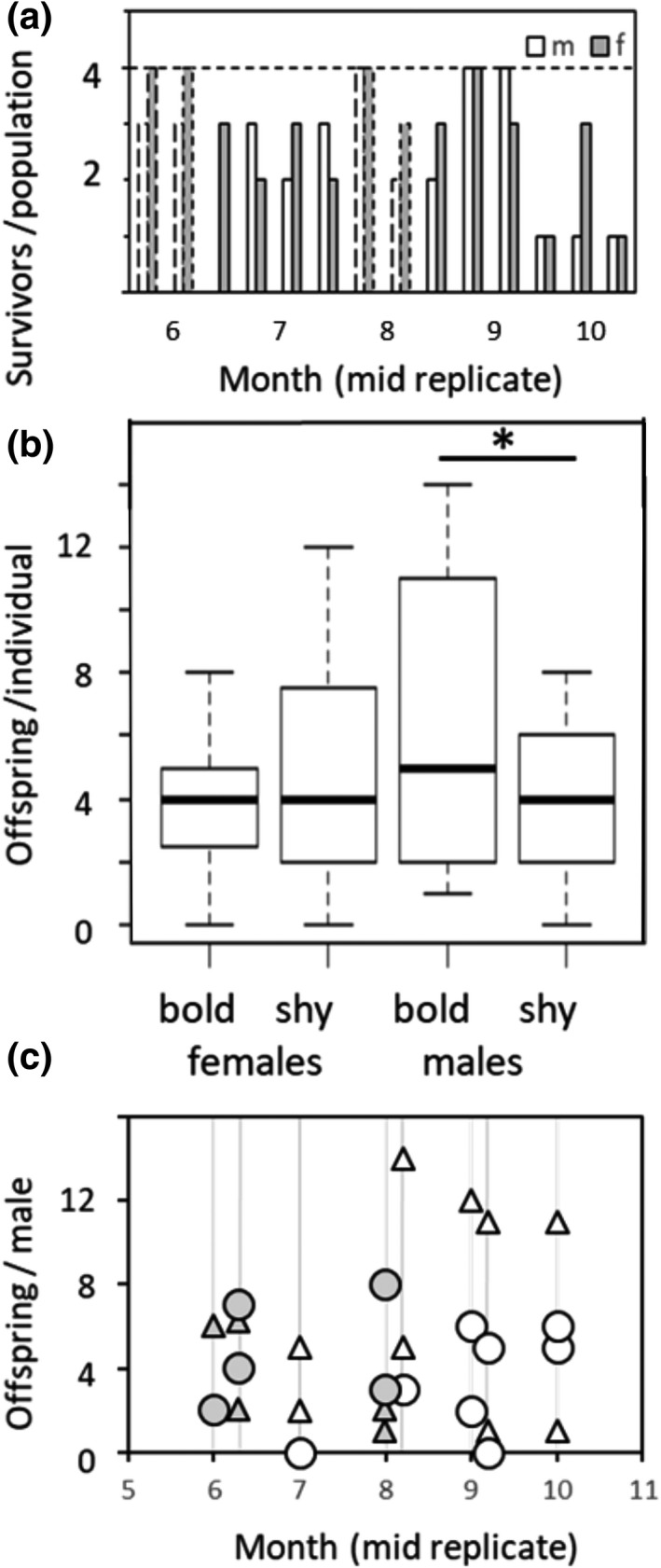
Fitness of common voles in experimental populations (a) survival of males and females (initial numbers: 4 each per population) in 14 populations over the season, year 1 solid lines, year 2 dashed lines, (b) number of assigned offspring per individual (8 populations, 57 parental candidates, 16–44 offspring per population assigned) at a significance level of *p* < .05 (asteriks), (c) number of assigned offspring per male, Δ bold male, ○ shy male, gray lines = 8 populations sorted by months, year 1 white, year 2 gray symbols

Daily home ranges (*n* = 1004 ranges, 58 animals) were highly repeatable within individuals (for *R*_adj_ = .44, CI: 0.32–0.54, *p* < .001). Range size was explained by an interaction of boldness type, sex, and experimental phase (three‐way interaction: home range χ^2^ = 22.9.8, *df* = 3, *p* < .001, effect sizes Table 3, Figure 3). To disentangle the three‐way interaction, we analyzed simple effects within different subsets. Among bold animals (*n* = 30 individuals of both sexes, *n* = 553 daily core areas), experimental phase affected day range size (χ^2^ = 23.9, *df* = 3, *p* < .001, Figure 3b) with smaller day ranges during the exploration phase (mean ± SD: 88% percent of respective population mean ± 45%), which differed from the first pregnancy phase (105% ± 55%) and the parturition and second mating phase (111% ± 55%) but not from the second pregnancy phase (98% ± 48%). Among shy animals (*n* = 28 individuals, *n* = 451 day ranges), sex differences varied between experimental phases (interaction χ^2^ = 22.1, *df* = 3, *p* = .001, Figure 3b). Within females, boldness type explained day range size depending on experimental phases (interaction: χ^2^ = 12.2, *df* = 4, *p* = .006, *n* = 31 females, *n* = 542 daily home ranges). Separate analyses of main effects within combinations of boldness types by sex revealed that shy females (*n* = 15 individuals, *n* = 271 daily home ranges) had larger day ranges during the exploration phase (106% ± 58%) and first pregnancy (108% ± 36%), and size decreased during later phases (mating: 92% ± 20%) and second pregnancy (79% ± 32%; experimental phase: χ^2^ = 54.0, *df* = 3, *p* < .001, Figure 3b). In contrast, bold females (*n* = 16 individuals, *n* = 272 daily home ranges) used smaller day ranges during exploration phase (86% ± 35%) compared to later phases (mean 95% 105%, experimental phase: χ^2^ = 17.5, *df* = 3, *p* < .001; Figure 3b). Across all phases, day ranges of males were larger (104% ± 40%) than those of females (94% ± 44%, χ^2^ = 7.2, *p* = .007, Table 4). Males’ (*n* = 27 individuals, n = 461 days) range sizes varied with experimental phase (χ² = 16.1, *df* = 3, *p* = .001) but not with boldness types (χ² = 0.1, *df* = 1, *p* = .789, interaction not significant). Males had the smallest day ranges during the exploration phase (87% ± 52%) compared to later phases (mean 103%–111%, Figure 4).

### 3.2 Fitness of animals

In total, 73 of 113 (65%) released common voles were recaptured from the enclosures. Females tended to survive better (73%, Table 2, Figure 4a, mean ± SD: 2.9 ± 1.0 per population) than males (57%, 2.3 ± 1.3 per population, χ^2^ = 2.8, *p* = .094), and survival tended to decrease with month from 88% survival in June replicates to 35% in October replicates in October, χ^2^ = 3.5, *p* = .060). Boldness type did not explain variation in survival (χ^2^ = 1.4, *p* = .242, Figure 4a, effect sizes in Table 2). During the time in the enclosures, animals gained on average 12.3 g body mass (mean ± SD body mass after the experiment: males 38.1 ± 6.5 g; females 32.7 ± 5.9 g, including gravid females).

Fifty‐seven experimental animals were parental candidates, and offspring were assigned to 51 of them (89%). The number of offspring assigned to an individual ranged from 0–14 and was predicted by an interaction of sex and boldness type (χ^2^ = 5.7, *p* = .017, effect sizes Table 2, Figure 4b). Follow‐up analysis within sexes (GLMM of offspring numbers, Poisson error distribution) showed that overall, bold males produced more offspring (5.6 ± 4.6, *n* = 14) than shy males (3.9 ± 2.5 offspring, *n* = 13, simple effects within males: χ^2^ = 4.4, *p* = .036, effect size ß = −0.38 ± 0.18, Figure 4c shows distribution of offspring per male within the 8 populations analyzed). In females, boldness types did not predict reproductive output (4.3 ± 3.1 offspring, χ^2^ = 1.4, *p* = .227, ß = −0.22 ± 0.18, *n* = 15 females per type, Figure 4b).

## 4 DISCUSSION

In experimentally created populations of known behavioral‐type composition, we were able to show that individual differences in boldness behavior covaried with risk taking, space use, and fitness under near natural conditions. We created experimental populations combining the opposite ends of the distribution of behavioral phenotypes (bold and shy animals) and studied behavior in the field with automated tracking methods. We found that behaviors measured in the field were consistent within individuals over time, quantifying themselves for animal personality traits. We further found that bold animals of both sexes visited the risky edges of the enclosures more frequently than shy animals of the same sex. In females, effects of boldness type were detected during limited times only (Figure 4). While males ranged over larger areas than females (as shown earlier for this species, e.g., Briner et al., 2005), range sizes differed between shy and bold females immediately upon release. Shy females apparently explored larger areas initially and then settled in smaller ranges, while the opposite pattern was observed in bold females and males of both behavioral phenotypes. Bolder males took higher risks and fathered more offspring than shy males. Boldness did not explain survival probability in both sexes, however. Mortality of voles tended to increase in autumn (Figure 2a), probably due to colder weather and decreasing quality of forage, mirroring annual population dynamics of common voles (Eccard & Herde, 2013).

### 4.1 Behavior and boldness types

Boldness as measured in many small mammals in laboratory settings using open field and exploration tasks may be a direct predictor of risk taking (Dammhahn & Almeling, 2012). The main source of mortality for voles is predation, rendering them a key species in natural food chains with many ground and avian predators preying on them (Halle, 1988; Jędrzejewski & Jędrzejewska, 1993; Norrdahl & Korpimӓki, 1995). Since boldness may be directly linked to mortality risk (Smith & Blumstein, 2008, but see Moiron et al., 2020), it should have a strong impact on spatial behavior, and the exposure to predators.

In the experimental populations in our study, bold males were taking higher risks by visiting the short vegetation edges of the enclosure more frequently than shy males. Boldness is often correlated with exploration at the phenotypic level, so bolder individuals were often reported to explore an area faster than shy individuals, which could result in a more superficial exploration and exploitation of resources (Mazza et al., 2018; Sih et al., 2004; Wolf et al., 2007a; Wolf et al., 2007b). In our study, we found some support for this pattern with bold males being registered in the risky area of the enclosure more often and for shorter periods than shy males, probably indicating a quicker and superficial exploration of these areas. The duration of single visits did not differ among males, but among females. Females visited the risky edge less frequently than males, and stayed very shortly at the passage counters during their first pregnancy, compared to the exploration phase and the second pregnancy (Figure 3). Since long stays indicated a slower and more careful passage, as indicated by our pilot experiment, we assume these phases are used for exploration (of the novel area, or to find a new nest for giving birth to the second litter), while during the first pregnancy females passed the counters quickly and on paths known to them. Females never appeared at the passage counters during the second mating phase, probably because voles mate briefly during a postpartum estrus and females may have spent this time close to their nests nursing new borne offspring.

Individual daily range sizes were highly repeatable over time, indicating intrinsic individual differences in space use. Similarly, home range and core area size, as well as microhabitat characteristics of bank voles (*Myodes glareolus*) and striped field mice (*Apodemus agrarius*)—automatically tracked under natural conditions—covaried with individual behavioral differences (Schirmer et al., 2020). Thus, overall among‐individual differences in space use may contribute to individual niche specialization (Pearish et al., 2013; Spiegel et al., 2017), facilitating the coexistence of similar species (Schirmer et al., 2020). The distribution of individuals in space and time is an important determinant for key aspects of the social system, for example, the mating system (Heckel & von Helversen, 2002, 2003; Lukas & Clutton‐Brock, 2013), and of foraging under risk. Hence, behavioral type‐specific space use should have consequences for survival and reproductive success.

Directly after transfer to the novel environment (exploration phase), bold females and both types of males in our experimental populations used smaller day ranges than at later stages of the experiment, indicating an initial reduction in mobility. Shy females used larger areas during the first three days, and settled in areas that later allowed them to maintain small home ranges (Figure S1). At first glance, this differs from established populations in different species where bold animals (of both sexes) had larger ranges than shy ones (rodents: Boon et al., 2008; Schirmer et al., 2020, birds: Minderman et al., 2010). Our finding is more in line with observations of shy animals being more thorough explorers in novel environments (Marchetti & Drent, 2000; Mazza et al., 2018; Mutzel et al., 2013; Veerbek et al., 1994), which might give them an advantage under changing and harsh environmental conditions.

### 4.2 Fitness and boldness types

As predicted, bold males overall sired more offspring than shy males, although not in all populations. In line with our results, boldness scales positively with reproductive success in many species (Collins et al., 2019; Dingemanse & Réale, 2005; Godin & Dugatkin, 1996; Reaney & Backwell, 2007; Scherer et al., 2020; Smith & Blumstein, 2008). Several nonexclusive mechanisms might explain fitness benefits of being bold. (1) Bolder males might take higher risks in roaming in space to find receptive females and/or defend receptive females more successfully to monopolize paternity (Ophir et al., 2008; Smith & Blumstein, 2008; Wolf et al., 2007a; Wolf et al., 2007b); indeed, in our study bolder males were detected more often in risky areas of the enclosures. Appearance in such areas is sometimes used to infer dispersal tendencies (Hahne et al., 2011) and may also indicate wider roaming areas (Schirmer et al., 2020; Ward‐Fear et al., 2018). (2) Females could have a preference for bolder males (Godin & Dugatkin, 1996). If boldness was selected for in males, we would expect males to generally be bolder than females (Schuett et al., 2010), which is not supported by our other studies on voles (*M. arvalis*: Eccard & Herde, 2013; Herde & Eccard, 2013; *Myodes glareolus*: Mazza et al., 2018; Schirmer et al., 2019). (3) Reproductive success of males may be primarily determined by dominance rank (Dewsbury, 1982; Ellis, 1995) rather than personality *per se*, but both traits can be highly entangled so that boldness may predict dominance. (4) Among‐individual variation in behavior could be part of a larger pace‐of‐life syndrome (Dammhahn et al., 2018; Réale et al., 2010) and covariation between these traits might be maintained by density‐dependent selection (Milles et al., 2022; Wright et al., 2019). Microtine voles, in most places, frequently and predictably undergo massive fluctuations in population density, which are accompanied by population‐level differences in behavioral type (Eccard & Herde, 2013) and social environmental conditions. Further, for short‐lived iteroparous animals in seasonally fluctuating environments, life history trajectories and social environmental conditions (e.g., density) are predictable (Eccard et al., 2017; Eccard & Herde, 2013). Selection may favor bolder behavioral types in high density and high competition phases of the yearly fluctuation cycle, which ought to express a more risk‐prone pace‐of‐life syndrome (Herde & Eccard, 2013). Shy behavioral types and risk‐averse pace‐of‐life syndrome may potentially be favored by higher survival at low densities in winter or early in the breeding season, and subsequently have a higher contribution to the increasing population in spring than bold individuals. In our data, in the replicates run earlier in the breeding season shy males had the highest reproductive success (Figure 2c), while later in the season, when in wild, natural populations densities would be high, reproductive success was skewed in favor of single, bold males. This may indicate a density‐dependent selection of different pace‐of‐life syndromes in fluctuating populations, triggered by seasonal cues. Future studies should test the relationships between social environment, predictable seasonal life history trajectories, animal personality, and fitness.

We did not detect an effect of boldness type on reproductive success in females. Thus, the fitness consequences of boldness might be sex‐specific, similar to the findings in black browed albatrosses (Patrick & Weimerskirch, 2014). Access to food and safety are major determinants of reproductive success in female mammals (Crook & Gartlan, 1966; Emlen & Oring, 1977; Lukas & Clutton‐Brock, 2013; Ophir et al., 2008; Terborgh & Janson, 1986); since common voles mainly eat grass and find shelter in underground burrows, our large grassland enclosures should not have provided a resource limited environment. Further, reproductive skew is generally lower in females than in males (Bateman, 1948) and once female voles reproduce, they usually produce entire litters. We expected bold females to occupy larger home ranges (as bank voles under natural conditions: Schirmer et al., 2019) and thus be able to provision their offspring better (e.g., as in blue tits: Mutzel et al., 2013) compared to shy females. However, differences in provisioning (lactation) would be difficult to detect among different types of female mammals in an outdoor study.

In contrast to our prediction but in line with results of a recent meta‐analysis (Moiron et al., 2020), survival did not differ between boldness types. The finding may be caused by the rather benign setting of our experiment in a favorable season, a low population density, and reduced predation pressure since ground predators were excluded. Alternatively, limited space might be another explanation, since male voles might roam larger areas under natural conditions than offered in our enclosures. Overall, the survival rate of common voles in our study (35% over 7 weeks) seemed high compared to those reported elsewhere: 2 to 9% daily mortality of voles with radio transmitters (field voles, East European voles and bank voles; Norrdahl & Korpimӓki, 1995), or 50% mortality over four weeks in agricultural fields (common voles; Jacob, 2003). In our experiment, survival dropped toward the end of the season (Figure 2a) for animals of any boldness type, when in wild populations peak densities would crash (Eccard & Herde, 2013) and the adult animals captured during summer would reach the end of their life span. Meanwhile, if in voles mortality would follow a disruptive viability selection, such as found in Eastern chipmunks (*Tamias striatus*), and both high and low extremes of behavioral types would had elevated survival compared to intermediate types (Bergeron et al., 2013), we would not be able to detect this pattern since we selected extreme boldness types from the ends of a behavioral gradient.

## 5 CONCLUSIONS

Overall, our results highlight that among‐individual differences in behavior translate into variation in space use, risk taking, and reproductive success in near‐natural populations. Reproduction was biased toward single bold males in late summer replicates. Since variation in boldness is maintained in natural populations, we assume that shy types may have fitness advantages in other seasons (Lonn et al., 2017) or at different population densities (Wright et al., 2019), which remains to be tested. With daily range sizes being highly repeatable within individuals, consistent individual space use patterns may facilitate individual niche specialization and thus affect within‐ and between‐species ecological interactions. We show with this experiment, that behavioral phenotypes covary with risk‐taking behavior in the field, and that behavioral differences are thus expressed in natural settings. We can further show that behavioral phenotypes are fitness relevant.

## CONFLICT OF INTEREST

Authors declare that there are no competing interests.

## AUTHOR CONTRIBUTIONS

**Jana A. Eccard:** Conceptualization (lead); Formal analysis (equal); Funding acquisition (lead); Methodology (equal); Resources (lead); Supervision (lead); Visualization (equal); Writing – review & editing (equal). **Antje Herde:** Conceptualization (equal); Data curation (lead); Formal analysis (equal); Investigation (equal); Methodology (equal); Visualization (equal); Writing – original draft (lead). **Andrea C. Schuster:** Data curation (supporting); Investigation (supporting); Methodology (supporting). **Thilo Liesenjohann:** Investigation (equal). **Tatjana Knopp:** Investigation (equal). **Gerald Heckel:** Formal analysis (supporting); Methodology (supporting); Resources (supporting); Writing – review & editing (supporting). **Melanie Dammhahn:** Formal analysis (supporting); Writing – review & editing (equal).

## Supporting information

Table S3

Supplementary Material

## Data Availability

Data are accessible on dryad https://doi.org/10.5061/dryad.44j0zpcfs.
