# Supplementary material for: Fitness, risk taking, and spatial behavior covary with boldness in experimental vole populations
Source: Ecol Evol. 2022 Feb 9;12(2):e8521. doi: 10.1002/ece3.8521 (PMC8829380; doi:10.1002/ece3.8521)
Supplement: Supplementary file 1 — Table S3 [file ECE3-12-e8521-s002.docx]

**Herde et al. Fitness, risk taking and spatial behaviour covary with animal personality in experimental vole populations - Supplement**

**Table S5:** Genotyping, Methodological details

| **Step** | **Method** |
| --- | --- |
| Storage of tissue samples | 100% Ethanol at -20% |
| Extraction | 2010: *NucleoSpin® Tissue Kit* (*MACHEREY-NAGEL,* Dueren, Germany) |
|  | 2011: *First DNA all-tissue DNA extraction Kit* (GENIAL GmbH, Troisdorf, Germany) |
| Elution | 50 to 250 µl sterile water depending on size of DNA pellet |
| Storage | DNA was stored at -20 C |
| Amplification | *QIAGEN® Multiplex PCR Kit* (*QIAgen*, Hombrechtikon, Switzerland). |
| PCR Primer sets (Braaker & Heckel 2009): | |
| set 1 for markers | *MM1, MM8, MSCRB5, MAG25, MAG06, Mar063, Mar113** |
| set 2 for markers | *MM6, Moe2, Mar016, Mar049, Mar80, AVPRin** |
| set 3 for markers | *Mar003, Mar076, Mar058** |
| PCR conditions | 1x *QIAGEN® Multiplex PCR Mastermix* |
|  | 0.2 µM of each forward and reverse primer |
|  | approx. 50 ng of DNA template (reaction volume 10 µl) |
|  | *PTC-100 Thermal Cycler (MJ Research Inc*., Watertown, USA): |
|  | thermal profile 95 C (5 min), 30 cycles of 94 C (30 sec), 57 C (90 sec), |
|  | 72 C (60 sec), final extension 60 C (30 min) |
| Dilution of PCR products | 20 µl sterile water |
| Denaturation | 1.2 µl of the diluted PCR product mixed with |
|  | *GeneScan™ 500 LIZ™ Size Standard (Applied Biosystems*, Foster City, USA) |
|  | denatured at 95 C for two minutes. |
| Genotyping (elektrophoresis) | *ABI PRISM® 3130xl Genetic Analyzer* (*Applied Biosystems*). |

No locus showed deviations from Hardy-Weinberg equilibrium, all parental candidates showing more than one mismatching allele within the 16 loci analysed in comparison with the respective offspring were removed, estimations of the critical difference in log-likelihood scores (ΔLOD) were performed by simulations generated by 100 000 simulated offspring. On average 99.3 ± 0.4 % of loci were typed and we run simulations with an estimated typing error rate of 1%. We conducted one to three simulations per population depending on number of population replicates within the respective population. For the first simulation we used following parameters: we entered all individuals of the inserted parental population and the captured offspring as potential parents. Thereby, we assumed a proportion of 90% sampled candidate parents. Into later simulations, we entered all individuals of the inserted parental population (also including the individuals of a previous population replicate in the same population) and all captured offspring at that time as potential parents, but we assumed a proportion of 80% sampled candidate parents. Furthermore, in case that more than one parent combination showed no allele mismatching, we did not accept the parentage identification based on likelihood assignment of *CERVUS*.
