# Supplementary material for: Fitness, risk taking, and spatial behavior covary with boldness in experimental vole populations
Source: Ecol Evol. 2022 Feb 9;12(2):e8521. doi: 10.1002/ece3.8521 (PMC8829380; doi:10.1002/ece3.8521)
Supplement: Supplementary file 2 — Supplementary Material [file ECE3-12-e8521-s001.docx]

Supplemental Material

Table SEM 1: Schedule of population replicates of 14 experimental common vole populations with 8 founder animals of selected behavioural phenotypes (bold or shy) over two years using 6 enclosures. Trapping refers to time period the founder animals were collected from wild populations, offspring numbers refer to captured, genotyped and assigned offspring (missing population replicates yielded <8 offspring), day ranges refer to the numbers of individual day ranges obtained by automated telemetry over 2 – 4 experimental phases (missing population replicates refer to enclosure with telemetry failures or information only on one experimental phase).

| **year** | **Trapping** | **Test cohort** | **Enclosure** | **Field season** | **Offspring** | **Day ranges** |
| --- | --- | --- | --- | --- | --- | --- |
| 1 | Apr-May | C1 | E1 | July-Aug |  |  |
|  |  |  | E8 | July-Aug | 16 |  |
|  | May-June | C2 | E2 | Aug |  | 129 |
|  |  |  | E7 | Aug | 44 |  |
|  | July-Aug | C3 | E3 | Sept | 40 | 227 |
|  |  |  | E6 | Sept | 34 |  |
|  | July-Sept | C4 | E1 | Sept-Oct | 42 | 24 |
|  |  |  | E8 | Sept-Oct |  | 77 |
|  | August-Sept | C5 | E2 | Oct |  | 103 |
|  |  |  | E7 | Oct |  |  |
| 2 | March-May | C6 | E2 | June | 28 | 147 |
|  |  |  | E7 | June |  | 80 |
|  | June | C7 | E1 | Aug | 16 | 87 |
|  |  |  | E8 | Aug | 38 | 130 |

Table SEM 2: Comparison across all replicates of common voles categorised as bold and shy in subsequent test cohorts


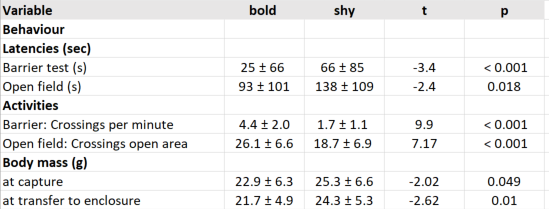


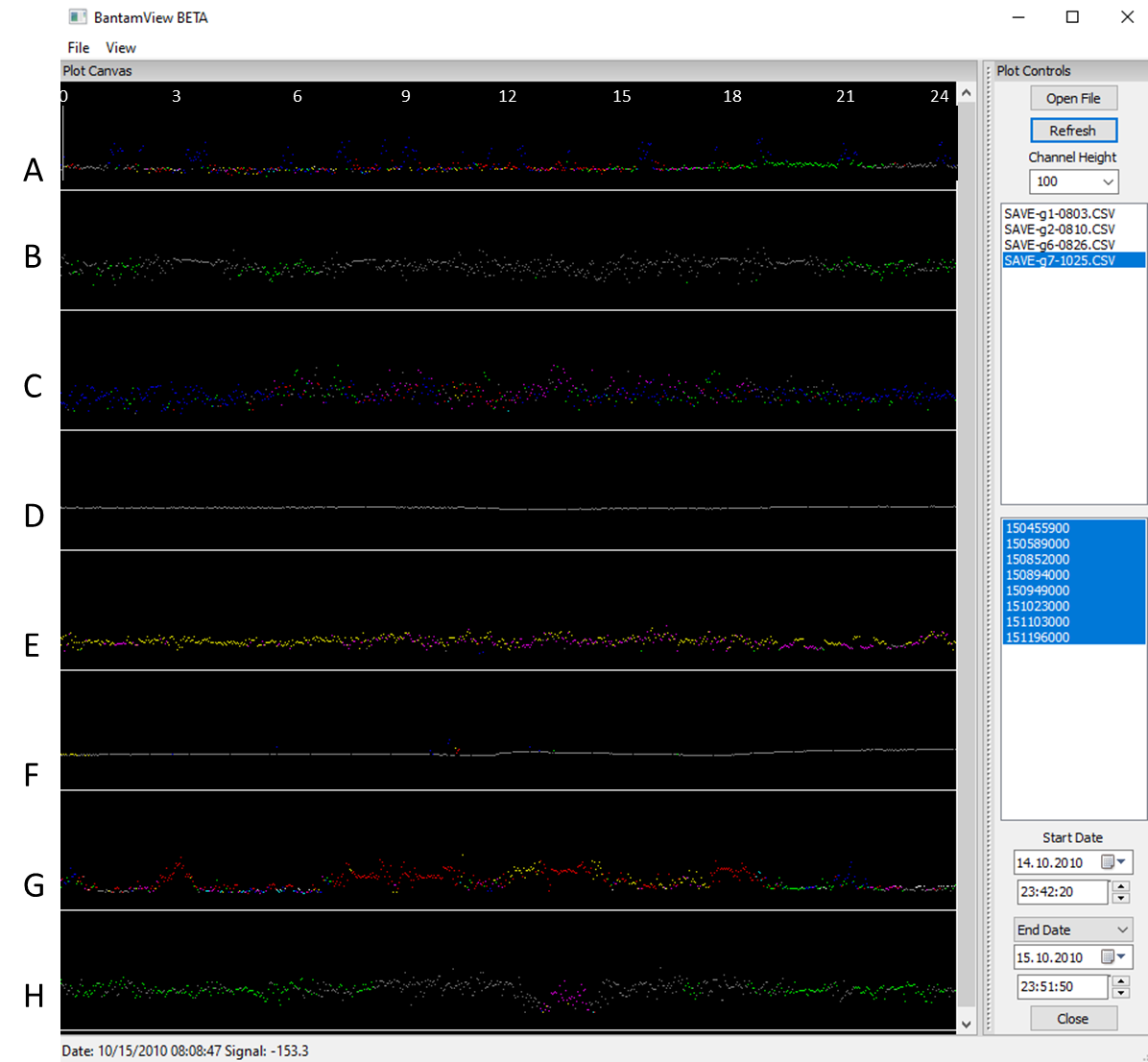


Figure S1: Diagnostic plots (Screenshot Bantam viewer, sparrowsystems.biz) showing relative signal strength (y achsis) and antenna ID (colour) capturing the highest signal strength in a 50mx50m telemetry grid surrounded by 8 antennas for eight VHF radiotags (frequencies displayed in the second box, right) over 24h (date and time low right corner, daytime along x achsis white numbers in top row). Tags were attached to common voles. Interpretation of plots: Tag A, B, C, D,. G and H showed regular changes in signal strength (y achsis) and/or in locations (change of colour indicates movement inside the grid) due to polyphasic activity of voles roughly every three hours, while Tags D and F produced a flat signal because the tag was immobile.
